# Moderating Effect of Insulin Resistance on the Relationship between Gray Matter Volumes and Cognitive Function

**DOI:** 10.3390/jcm7110413

**Published:** 2018-11-04

**Authors:** Jiyeon Lee, Jihyeon Kim, Seong A Shin, Soowon Park, Dong Hyun Yoon, Hongrae Kim, Yu Kyeong Kim, Min Kyong Moon, Bo Kyung Koo, Jun-Young Lee

**Affiliations:** 1 Department of Psychiatry, SMG-SNU Boramae Medical Center, Seoul National University College of Medicine, Seoul 07061, Korea; pavv_@naver.com (J.L.); kimkimseven@naver.com (H.K.); 2 College of Medicine, Seoul National University, Seoul 03080, Korea; jane.jihyeonkim@gmail.com; 3 Department of Biomedical Sciences, Seoul National University, Seoul 08826, Korea; sshi082@gmail.com; 4 Department of Education, Sejong University, Seoul 05006, Korea; spark@sejong.ac.kr; 5 Department of Psychiatry and Behavioral Science, SMG-SNU Boramae Medical Center, Seoul 07061, Korea; ycool14@snu.ac.kr; 6 Department of Nuclear Medicine, SMG-SNU Boramae Medical Center, Seoul 07061, Korea; yk3181@snu.ac.kr; 7 Department of Internal Medicine, Seoul National University College of Medicine & SMG-SNU Boramae Medical Center, Seoul 07061, Korea; mkmoon@snu.ac.kr (M.K.M.); bokyungkoomd@gmail.com (B.K.K.); 8 Department of Psychiatry and Neuroscience Research Institute, Seoul National University College of Medicine & SMG-SNU Boramae Medical Center, Seoul 07061, Korea

**Keywords:** Alzheimer’s disease, Insulin Resistance, Cognitive Decline, Gray Matter

## Abstract

Background: It is controversial whether exposure to insulin resistance accelerates cognitive deterioration. The present study aimed to investigate the association between insulin resistance and gray matter volume loss to predict the cognitive decline. Methods: We recruited 160 participants (78 with Alzheimer’s disease and 82 without Alzheimer’s disease). Insulin resistance, regional gray matter volume, and cognitive function were assessed. A hierarchical moderated multiple regression (MMR) model was used to determine any associations among insulin resistance, structural changes in the brain, and cognitive decline. Results: The volumes of 7 regions in the gray matter were negatively related to insulin resistance in Alzheimer’s disease (*p* =0.032). Hierarchical MMR analysis indicated that insulin resistance did not directly affect the cognitive decline but moderated the cognitive decline through the decrease in gray matter volume in the key brain regions, i.e., inferior orbitofrontal gyrus (left), middle cingulate gyrus (right), hippocampus (right), and precuneus (right) (*p* < 0.05 in each case). Conclusion: Insulin resistance appears to exacerbate the cognitive decline associated with several gray matter volume loss.

## 1. Introduction

Insulin resistance is associated with obesity, hyperlipidemia, chronic low-grade inflammation, and low levels of physical activity, which is hallmark of the metabolic syndrome and type 2 diabetes mellitus [1,2]. Insulin resistance is a major feature of type 2 diabetes mellitus, whereas type 1 is due to damage of pancreatic B-cells of Langerhans islets and loss of insulin [3]. Insulin signaling dysfunction has been found to inversely affect several neural processes, and may accelerate brain atrophy in Alzheimer’s disease (AD) [4]. It has been reported that patients with AD have high insulin resistance, which indicates increased insulin levels and reduced binding efficacy on neuronal synapses and astrocytes [5]. Brain insulin resistance might be a feature of diabetes mellitus and is emerging as a potentially important feature of AD. Some investigators refer to AD as type 3 diabetes, or an insulin resistant brain state, because AD is closely related to dysfunction of both insulin signaling and glucose metabolism in the brain [6].

Few studies have assessed the relationship between insulin resistance and brain atrophy. For regional gray matter volume, clinical studies have almost focused on the hippocampus. Studies have demonstrated a significant negative relationship between insulin resistance and the right or total hippocampal volume, as well as overall cognitive performance [7,8]. One study suggests that insulin resistance is associated with atrophy in regions affected by early AD in middle-aged cognitively normal participants [9]. Although the effects of insulin resistance on the brain volume are not well characterized, studies have demonstrated that insulin resistance is significantly associated with brain atrophy.

Inconsistent findings between insulin resistance and cognitive decline have been reported. Insulin resistance is found to accelerate cognitive decline and increase AD risk [10,11]. Peripheral insulin resistance is often associated with cognitive decline in middle-aged and elderly individuals [12,13,14,15], mild cognitive impairment (MCI) patients, and patients with accelerated conversion of MCI to AD [16,17]. However, a few studies have shown that insulin resistance or insulin-related variables are not associated with cognitive performance [18,19,20].The inconsistent results suggest that insulin resistance act as a moderator rather than a direct effector of cognitive decline. Therefore, further studies are needed to investigate the causative relationship between insulin resistance and cognitive impairment or brain volume. 

The major aim of this study was to investigate the relationship of insulin resistance with structural changes of brain and cognitive decline. We hypothesized that insulin resistance may induce the atrophy of gray matter volume, especially related with AD, and play a moderating role between brain atrophy and cognitive decline. However, insufficient and inconsistent findings on the specific role of insulin resistance hinder the formulation of any definitive hypotheses.

## 2. Material and Methods

### 2.1. Participants

A total of 160 participants were recruited at the dementia clinic at Seoul Metropolitan Government-Seoul National University Boramae Medical Center of South Korea. These participants were aged ≥65 years. AD was diagnosed by psychiatrists according to the probable or possible AD criteria of National Institute of Neurological and Communication Disorders and Stroke/Alzheimer’s Disease and Related Disorders Association [21].

Participants were eligible for inclusion in this study if they could read Korean, were community-dwelling, were free of comorbid conditions that could have affected cognition at baseline, and were capable of performing the Consortium to Establish a Registry for Alzheimer’s disease (CERAD) battery. Exclusion criteria included no reliable informant, structural abnormalities on brain imaging, a history of other neurological disorders or severe physical illnesses that may affect the cognitive function, and a history of alcohol or drug abuse. All participants were invited to undergo whole-body magnetic resonance imaging (MRI). Nine MRI head scans were excluded from evaluation because of low image quality with artifacts.

This study was approved by the Institutional Review Board of Seoul National University Hospital and written informed consent was obtained from all participants.

### 2.2. Measures

Demographic data (i.e., sex, age, and education) were collected from each participant. In addition, basal glucose and insulin levels were measured, and the Korean version of Consortium to Establish a Registry for Alzheimer’s disease Assessment Packet (CERAD-K) was used to assess the cognitive function of participants.

#### 2.2.1. Insulin Resistance and Homeostasis Model Assessment of Insulin Resistance

The participants fasted for 12 hours before blood collection. Baseline fasting blood samples were drawn, and the fasting duration was recorded. Serum levels of total cholesterol, high-density lipoprotein (HDL) cholesterol, triglycerides, glucose, hemoglobin A1c (HbA1c), and insulin were evaluated.

A well-validated index of insulin resistance (homeostasis model assessment of insulin resistance, HOMA-IR) was calculated using fasting plasma glucose and insulin levels to assess the degree of insulin resistance [22]. HOMA-IR (Homeostatic Model Assessment for Insulin Resistance) is calculated as follows:

HOMA-IR = (fasting plasma insulin (μIU/mL) × fasting plasma glucose (mg/dL) × 0.0555)/22.5.

Insulin resistance plays a major role in the development of diabetes, and the presence of insulin resistance precedes the onset of the disease by 10–20 years [23,24]. Insulin resistance is also known as the best predictor of diabetes in individual [23,24]. HOMA-IR is used to quantify insulin resistance rather than merely identify the presence of diabetes.

#### 2.2.2. Cognitive Assessment

Each participant in this study was assessed by psychiatrists with advanced training in dementia according to CERAD-K (the Korean version of Consortium to Establish a Registry for Alzheimer’s Disease) clinical assessment battery, which is developed and validated for the Korean elderly populations [25].

The CERAD-K battery provides a reliable estimation of cognitive function in normal aging and AD. The total score of the CERAD-K neuropsychological battery range from 0 to 100.The CERAD-K battery consists of the following subtests: 15-item Boston naming test (15 points), word list memory (30 points), word list recall (10 points), word list recognition (10 points), constructional praxis (11 points), and verbal fluency (24 points).Mini-Mental State Examination (MMSE), designed to screen cognitive impairment, is a neurocognitive test with a score range from 0 to 30, and a higher score indicates better cognition. This study used the Korean version of MMSE, which is composed of tests on orientation to time and place (10 points), registration (3 points), recall (3 points), attention (5 points), repetition (1 point), language (2 points), and complex commands (6 points) [26].

### 2.3. Brain Imaging Analysis

A total of 160 participants underwent structural MRI (3 Tesla, Achieva, Philips Healthcare, the Netherlands). The acquisition parameters for structural T1 imaging were listed as follows: repetition time, 9.9 ms; echo time, 4.6 ms; voxel size, 1.00 × 0.98 × 0.98 mm. Structural images were preprocessed for voxel-based morphometric analysis using Statistical Parametric Mapping 8 (SPM8; Wellcome Department of Imaging Neuroscience, UCL, UK, https://www.fil.ion.ucl.ac.uk/spm) implemented in Matlab (2014a, The MathWorks, Inc., Natick, MA, USA). The gray matter images were segmented and normalized into a standard space using diffeomorphic anatomical registration using exponentiated lie algebra algorithms and tissue probability maps in the SPM8 software. Subsequently, the images were modulated to preserve tissue volume after warping and were finally smoothed with an isotropic Gaussian kernel of 10 × 10 × 10 mm at full-width at half-maximum. Using MarsBaR (version 0.43, Cambridge, UK) and AAL116 (Automated Anatomical Labelling) atlas, gray matter volumes in cortical and subcortical regions were extracted.

### 2.4. Statistical Analyses

Statistical Package for the Social Sciences 18 (SPSS Inc., Chicago, IL, USA) was used for data analysis, and α was set at 0.05.

Initially, to obtain the key brain regions, multivariate analysis of covariance (MANCOVA) and the generalized linear model were used. Subsequently, features like age, sex, education, and total intracranial volume were used as covariates. The hierarchical moderated multiple regression was used to assess the link between moderator (M, insulin resistance), predictors (X, regional brain volumes), and clinical results (Y, cognition).

## 3. Results

### 3.1. Demographic and Clinical Characteristics of Participants

The demographic and clinical characteristics of the study population are shown in Table 1. The included 160 participants in this study had a mean age of 74 ± 6.74 years with 28.9% males and 67.5% females. Most participants did not have a history of diabetes (68.1%) and were not obese (body mass index < 25 kg/m^2^; 68.75%). The mean HOMA-IR score (2.63 ± 1.96) was low. In addition, 34.38% of participants had above high school education (45.8%). Mean levels of triglycerides, low-density lipoprotein and HDL cholesterol, and total cholesterol were normal. The mean MMSE and CERAD scores were 21.13 ± 5.51 and 48.59 ± 17.24, respectively.

### 3.2. Structural Changes in the Brain Associated with Insulin Resistance Exposure

In order to determine the key brain regions that are affected by insulin resistance in AD, MANCOVA with the generalized linear model was performed (control individuals with high insulin resistance vs. AD with low insulin resistance). Only regions with *p* < 0.1 were selected. If either of the right or left regions was significant, both regions were included (Table 2).

Statistically significant insulin resistance-related reduction was demonstrated in sevenregions: right orbitofrontal gyrus (*b* = −0.018, *p* = 0.027), left middle cingulate (*b* = −0.019, *p* = 0.009), right middle cingulate (*b* = −0.013, *p* = 0.046), right posterior cingulate gyrus (*b* = −0.014, *p* = 0.025), right hippocampus (*b* = −0.014, *p* = 0.019), right parahippocampus (*b* = −0.017, *p* = 0.036) and left precuneus (*b* = −0.01, *p* = 0.049). In a 90% confidence interval, additional 5 areas were also included: the left orbitofrontal gyrus (*b* = −0.012, *p* = 0.052), right anterior cingulate gyrus (*b* = −0.012, *p* = 0.091), left posterior cingulate gyrus (*b* = −0.015, *p* = 0.081), left hippocampus (*b* = −0.01, *p* = 0.071), and right precuneus (*b* = −0.009, *p* = 0.058).

### 3.3. Moderating Effects of Insulin Resistance on the Relationship between Neuropathological Variations in Key Brain Regions Sensitive to Insulin and Cognitive Deterioration

Table 3 shows the control and moderating effect of the insulin resistance on brain structure-related cognitive deterioration.

Age (*b* = −0.140, *p* = 0.004) and education (*b* = 0.629, *p* < 0.001) could be used to predict the cognitive function in the initial phase. The value of explanatory power of control variables was 30.6% (*p* < 0.001). In the second phase, the right orbitofrontal gyrus (*b* = 21.655, *p* = 0.053), left anterior cingulate gyrus (*b* = 18.026, *p* = 0.025), right anterior cingulate gyrus (*b* = 20.131, *p* = 0.005), left middle cingulate gyrus (*b* = 63.659, *p* = 0.059), and right parahippocampal gyrus (*b* = 31.469, *p* = 0.079) were significantly related to cognitive function. The explanatory power that cognitive function is predictable by brain structure was 12.3% (*p* < 0.012).

HOMA-IR (*b* = 0.706, *p* = 0.706) and the decision coefficient had no statistical relevance in the third phase. In the final phase, the analysis of the adjustment effect revealed the interaction between the brain volume and HOMA-IR (as a moderator). After applying the adjustment effect to the model, the cognitive function explanation power increased to up to 52.6% by a margin of 9.6% (*p* = 0.042). Table 3 shows the results of the interactions of HOMA-IR with several brain regions: the left orbitofrontal gyrus (*b* = −160.049, *p* = 0.014), right middle cingulate gyrus *(b* = −293.304, *p* = 0.047), right hippocampus (*b* = −216.084, *p* = 0.099), and right precuneus (*b* = −98.985, *p* = 0.095).

## 4. Discussion

In this study, separate voxel-wise analyses indicate that HOMA-IR acts as a moderator between decreased gray matter, affected by insulin resistance in AD, and cognitive decline, although it cannot directly predict cognitive decline. Therefore, HOMA-IR is a pure moderator, rather than a direct predictor, on the relationship between structural changes in the brain and cognitive decline. Most studies to date have focused on the direct association between insulin resistance and cognitive decline or brain atrophy [9,16,27,28]. Our study is meaningful because it reveals the moderating effect of insulin resistance on the relationship between gray matter volumes and cognitive function.

Based on previous studies, insulin receptors are expressed in the astrocytes and neurons and are densely distributed throughout the brain, including the olfactory bulbs, cerebral cortex, hippocampus, hypothalamus, amygdala, and septum [29,30]. In our study, regions, including the orbital part of the inferior frontal gyrus, cingulum, hippocampus, and precuneus, were shown as the candidates of insulin-sensitive key brain areas. These regions are associated with insulin-related changes in central nervous system (CNS) pathophysiology. Among these, four regions (the orbitofrontal gyrus, right middle cingulate gyrus, right hippocampus, and right precuneus) had significant interaction with insulin resistance, resulting in cognitive decline. The results are partly consistent with those in previous studies, which indicate that insulin resistance is associated with low volumes of the orbitofrontal cortex, middle cingulate, and precuneus and has similar patterns in patients with very early MCI up to early AD [9,31] and that the increased insulin response is positively associated with longitudinal brain volume in non-diabetic AD subjects [32]. 

The fundamental mechanisms underlying the association between insulin resistance, decreased gray matter volume, and cognitive performance are largely unclear, and further investigation might explain the association. We incorporated various risk factors in the hierarchical moderated multiple regression models, and the findings suggest a moderating (rather than direct) effect of insulin resistance on the relation between decreased brain volume and cognitive decline. The results indicate that insulin resistance, accompanied by changes in insulin-sensitive brain regions, might accelerate cognitive decline. However, it is important to note that no definite evidence indicates that brain atrophy relates to specific etiologies; therefore, further studies are needed to evaluate the association between insulin resistance and cognitive decline.

**Figure 1:**
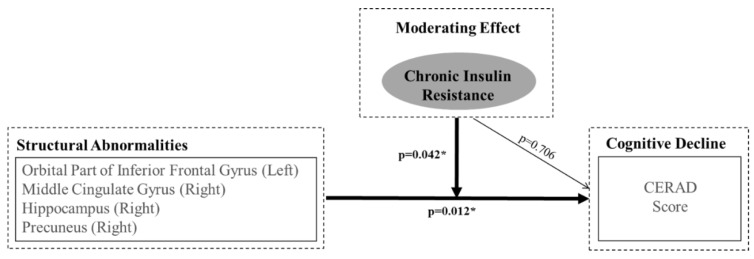
Representation of Hierarchical Moderated Multiple Regression. The orbital area of inferior the frontal gyrus (left), the middle cingulate gyrus (right), the hippocampus (right), and the precuneus (right) are regions where insulin resistance has a moderating effect between structural changes and cognitive deterioration.

Many previous studies have investigated the relationship between diabetes-associated factors (such as HbA1c, diabetes status, blood glucose, cognitive function, and brain atrophy and/or metabolism), in addition to insulin resistance and cognitive impairment [33,34,35]. The current study indicates that only insulin resistance has a significant effect on the relationship between structural changes in key brain regions sensitive to insulin and cognitive deterioration. The present results suggest that insulin resistance is associated with cognitive decline, but HbA1c or blood glucose is not (Figure 1).

Insulin, a peptide secreted by pancreatic beta cells, enters the CNS by penetrating the blood-brain barrier. The brain insulin signaling plays critical roles in regulating food intake, body reproduction, and learning and memory [36] and is essential to proper synaptic metabolism, protein synthesis, and neuronal survival. Chronic hyperinsulinemia leads to the downregulation of insulin receptors located in the blood-brain barrier, thus resulting in brain insulin resistance, which might cause neurodegeneration. Disruption of the insulin signaling makes renders neurons vulnerable to metabolic stress, resulting in acceleration of neuronal dysfunction. Defects in the insulin receptor signaling are reported to be associated with decreased cognitive function and the development of dementia, including AD [37]. Loss of glial cells and axons, white matter rarefaction and shrinkage, and arteriolosclerosis may cause changes in the brain volume. Therefore, imaging and histopathological findings should be integratedly analyzed to better understand the pathological process of the effect of insulin resistance on brain atrophy.

This study has several limitations. First, the sample size was relatively small. This limitation may lead to a low statistical power and reduced chance of detecting a true interaction. Second, not enough information is available on the duration of diabetes mellitus or the diet pattern known to be associated with cognitive impairment. Third, the analyses in this study were cross-sectional. Therefore, this study could not examine possible associations between insulin resistance and changes in brain volumes over time. Future studies should use functional MRI to investigate the impacts of neural networks and their clinical significance in the analysis of the white matter. Thus, more information about the association between AD and insulin resistance would be obtained if positron emission tomography imaging with the 11C-Pittsburgh Compound-B ligand is used in future studies. Despite the limitations of this study, our findings showed its role as a moderator of insulin resistance, different from previous research. The study also suggests a link between insulin resistance, cognitive function and brain atrophy, which is a meaningful finding.

## 5. Conclusion

Only insulin resistance has a moderating effect on the association between volume loss of several gray matter regions and cognitive decline. Further investigation is needed to clarify the interaction between insulin resistance, brain atrophy, and cognitive function.

## Figures and Tables

**Table 1:** Demographic and Clinical Characteristics.

Categories	Mean (Standard Deviation or Number (%))
Age (years)	74.28 (6.74)
Sex (male/female)	48/112 (28.9/67.5)
Education (years)	7.87 (4.95)
Diabetes Mellitus	
DM (*N*, %)	51 (30.7)
Prediabetes (*N*, %)	80 (48.2)
Non-DM (*N*, %)	29 (17.5)
HbA1c (%)	6.24 (1.03)
Fasting Blood Sugar (mg/dL)	112.18 (31.96)
Fasting Insulin (mIU/L)	9.27 (5.79)
HOMA-IR	2.63 (1.96)
Total cholesterol (mg/dL)	181.9 (38.89)
HDL-cholesterol (mg/dL)	50.41 (16.86)
Triglycerides	122.69 (75.04)
Dementia	
Non-Dementia	82 (50.25)
AD	78 (48.75)
MMSE	21.13 (5.51)
CERAD	48.59 (17.24)
TIV (VBM)	1.56 (0.13)

Note: Data are presented as mean (standard deviation) for continuous variables and number (%) for categorical variables. DM, diabetes mellitus; HbA1c, hemoglobin A1c; HOMA-IR, homeostasis model assessment of insulin resistance; HDL, high density lipoprotein; AD, Alzheimer’s disease; MMSE, mini mental state examination; CERAD, consortium to establish a registry for Alzheimer’s disease; TIV, total intracranial volume; VBM, voxel-based morphometry.

**Table 2:** Structural changes relying on insulin resistance exposure in Alzheimer’s disease.

Dependent Variable	*b*	*t*	*p*
Brain Region			
Left orbital part of inferior frontal gyrus	−0.012	1.979	0.052
Right orbital part of inferior frontal gyrus	−0.018	2.256	0.027
Left anterior cingulate gyrus	−0.006	0.907	0.367
Right anterior cingulate gyrus	−0.012	1.714	0.091
Left middle cingulate	−0.019	2.705	0.009
Right middle cingulate	−0.013	2.035	0.046
Left posterior cingulate gyrus	−0.015	1.770	0.081
Right posterior cingulate gyrus	−0.014	2.286	0.025
Left hippocampus	−0.010	1.831	0.071
Right hippocampus	−0.014	2.393	0.019
Left parahippocampal gyrus	0.079	0.910	0.366
Right parahippocampal gyrus	−0.017	2.139	0.036
Left precuneus	−0.010	2.000	0.049
Right precuneus	−0.009	1.928	0.058

Note：MANCOVA with generalized linear model was done by using age, education, gender, and total intracranial volume as covariates.

**Table 3:** Analysis for moderating effect of insulin resistance by the hierarchical moderated multiple regression model.

Coefficients	*R^2^*	*b*	*β*	*p*
(constant)	0.526	32.056		0.031
Age		−0.126	−0.155	0.017
Gender		−0.184	−0.015	0.961
Education		0.614	0.553	<0.001
Total intracranial volume		−3.540	−0.084	0.774
Left orbital part of inferior frontal gyrus		24.130	0.163	0.249
Right orbital part of inferior frontal gyrus		28.515	0.176	0.043
Left anterior cingulate gyrus		−3.883	−0.031	0.181
Right anterior cingulate gyrus		20.603	0.163	0.017
Left middle cingulate		97.188	0.608	0.017
Right middle cingulate		81.576	0.532	0.020
Left posterior cingulate gyrus		18.788	0.120	0.396
Right posterior cingulate gyrus		−24.494	−0.145	0.797
Left hippocampus		8.727	0.080	0.468
Right hippocampus		77.891	0.669	0.039
Left parahippocampal gyrus		7.829	0.074	0.272
Right parahippocampal gyrus		60.357	0.554	0.014
Left precuneus		15.296	0.088	0.087
Right precuneus		27.050	0.166	0.221
HOMA-IR		0.895	0.067	0.471
(Left orbital part of inferior frontal gyrus) × (HOMA-IR)		−160.049	−0.445	0.014
(Right orbital part of inferior frontal gyrus) × (HOMA-IR)		−97.260	−0.219	0.316
(Left anterior cingulate gyrus) × (HOMA-IR)		−7.900	−0.031	0.489
(Right anterior cingulate gyrus) × (HOMA-IR)		−115.485	−0.364	0.281
(Left middle cingulate) × (HOMA-IR)		−161.328	−0.532	0.177
(Right middle cingulate)×(HOMA-IR)		−293.304	−0.881	0.047
(Left posterior cingulate gyrus)×(HOMA-IR)		0.849	0.002	0.489
(Right posterior cingulate gyrus)×(HOMA-IR)		11.730	0.033	0.422
(Left hippocampus) × (HOMA-IR)		125.596	0.413	0.414
(Right hippocampus) × (HOMA-IR)		−216.084	−0.671	0.099
(Left parahippocampal gyrus) × (HOMA-IR)		−24.386	−0.085	0.518
(Right parahippocampal gyrus) × (HOMA-IR)		−133.345	−0.430	0.654
(Left precuneus) × (HOMA-IR)		−65.383	−0.142	0.366
(Right precuneus) × (HOMA-IR)		−98.985	−0.264	0.095

Note. Hierarchical moderated multiple regression models were done by using age, education, gender, and total intracranial volume as covariates.HOMA-IR, homeostasis model assessment of insulin resistance.
